# High-risk factors of parotid lymph node metastasis in nasopharyngeal carcinoma: a case-control study

**DOI:** 10.1186/s13014-016-0691-x

**Published:** 2016-09-01

**Authors:** Hong-zhi Wang, Cai-neng Cao, Jing-wei Luo, Jun-lin Yi, Xiao-dong Huang, Shi-ping Zhang, Kai Wang, Yuan Qu, Jian-ping Xiao, Su-yan Li, Li Gao, Guo-zhen Xu

**Affiliations:** Department of Radiation Oncology, Cancer Hospital, Chinese Academy of Medical Science, Peking Union Medical College, No.17 Panjiayuan Nanli, Chaoyang District, Beijing, 100021 China

**Keywords:** Nasopharyngeal carcinoma, Periparotid lymph node metastasis, Risk factors, Case-control study

## Abstract

**Background:**

Although parotid-sparing IMRT decreased the dose distribution of parotid, parotid region recurrence has been reported. Prophylactic irradiation in parotid area would be necessary in patients with high risk of parotid lymph node metastasis (PLNM). This study was to detect the high-risk factors of PLNM in nasopharyngeal carcinoma.

**Methods:**

This was a 1:2 case-control study. All patients in this study were newly diagnosed NPC with N2-3 classification from January 2005 to December 2012. Cases were 22 sides with ipsilateral PLNM. Controls were 44 patients who were randomly selected from N2-3 disease in database.

**Results:**

20/1096 (1.82 %) NPC patients were found PLNM. Sum of the longest diameter for multiple lymph nodes (SLD) in level II was larger in case group than that in control group (6.0 cm *vs*. 3.6 cm, *p* = 0.003). Level II lymph node necrosis, level Va/b involvement, and rare neck areas involvement were more common in case group (*p* = 0.016, *p* = 0.034, and *p* < 0.001, respectively). RPN, level III, and level IV metastases showed no significant difference between the two groups. Multivariate analysis in logistic regression showed that only SLD ≥5 cm in II area (OR = 4.11, *p* = 0.030) and rare neck areas involvement (OR = 3.95, *p* = 0.045) were associated with PLNM in NPC patients.

**Conclusions:**

PLNM was an uncommon event in NPC patients. SLD ≥5 cm in level II and involvement in rare-neck areas may be potentially high-risk factors for PLNM. Sparing parotid in IMRT was not recommended for NPC patients with high risks of PLNM.

## Background

Nasopharyngeal carcinoma (NPC) is usually presented with regional lymph node metastasis. And retropharyngeal nodes and level II were the most commonly involved regions, with the incidence of 69 % and 70 %, respectively [1]. However, parotid lymph nodes (PLNs) were rarely involved.

In 2013, DAHANCA, EORTC, HKNPCSG, NCIC CTG, NCRI, RTOG, TROG consensus guidelines regarding the delineation of the neck node levels for head and neck tumors has defined the parotid lymph node group as level VIII, which included the subcutaneous pre-auricular nodes, the superficial and deep intraparotid nodes, and the subparotid nodes [2]. In anatomy, parotid lymph node receive efferent lymphatic from the frontal and temporal skin, the eyelids, the conjunctiva, the external auditory canal, the tympanum, the nasal cavities, the root of the nose, the nasopharynx, and the Eustachian tube. Theoretically, nasopharyngeal carcinoma is at risk of harboring parotid lymph node metastasis (PLNM). However, the reported incidence of PLNM in NPC patients was only 0.6–3.0 % [3–7]. Direct lymphatic drainage from nasopharynx to parotid was to be questioned.

Radiotherapy is the mainstay of definitive treatment modality for nasopharyngeal carcinoma. In conventional two-dimensional treatment era (Ho’s technique), lateral opposing fields encompassed the parotids in the irradiation volume. Mean dose of parotid was 55.3Gy [8] and ≥ grade II late xerostomia was 29.7 % [9]. With the development of radiation technique, parotid-sparing IMRT has decrease the dose distribution of parotid [10, 11] and improved patients’ quality of life, particularly xerostomia recovery [12]. However, with the use of parotid-sparing IMRT, parotid region recurrence has been reported. In 2007, Luo et al. [13] has showed 3 cases of parotid recurrence in NPC patients after IMRT. In 2013, Cao et al. reported 10 cases of periparotid recurrence and the incidence of parotid recurrence was 1.4 % [14]. Periparotid recurrence may be supposed to be related to sparing parotid in IMRT.

Although parotid lymph node metastasis is an uncommon event in NPC patients, and the target definition and coverage for patients treated with IMRT for parotid sparing is adequate, questions remain pertaining to what high-risk factors would lead to parotid lymph node metastasis in NPC patients and whether the target definition of parotid sparing should be adjusted in patients with high risks. To address these issues, we conducted this case-control study.

## Patients and methods

### Patients

We retrospectively reviewed the archives of 1096 patients with NPC who underwent IMRT in Cancer Hospital of Chinese Academy of Medical Science from January 2005 to December 2012. Twenty (1.8 %) patients were shown parotid lymph nodes metastasis when newly diagnosed. And 3 patients were found simultaneously bilateral parotid lymph node involvement. Of the 20 PLNM patients, 10 patients were diagnosed with fine needle aspiration (FNA) and cytological biopsy. And the other 10 patients were diagnosed with contrast-enhanced MR imaging and Doppler ultrasonography. According to the 7^th^ edition of American Joint Committee on Cancer (AJCC) staging system, 19/20 (95.0 %) patients were N2-3 classification, which was significantly higher than 616/1096 (56.2 %) in whole NPC patients (*p* < 0.001). However, in the subset of the N2-3 NPC patients, the rate of PLNM was only 3.3 %. N classification alone was not enough to select the high-risk PLNM patients.

### Study design

This was a 1:2 case-control study. All patients in this study were newly diagnosed NPC with N2-3 classification. One PLNM patients was removed because of the N1 disease. Case group was designed as the ipsilateral neck with PLNM, including 22 cases (totally 19 patients and 3 patients with bilateral PLNM). 44 NPC patients were randomly selected from the N2-3 patients without PLNM. And the control group was designed as the ipsilateral neck with primary tumor center, or the side with heavier neck disease. This study was performed after approval by the institutional review board and ethics committee. And informed consents from each participant were obtained.

MR imaging was performed in all patients. And the images were separately reevaluated by two radiologists and one radiation oncologist. The node level was divided according to the updated consensus guidelines in 2013 [2]. As the 3^rd^ echelon of draining nodes in NPC [1], we regarded the level Ia/b, Vc (the lateral supraclavicular nodes), and VIa/b as the rare-neck areas. Information of the regional lymph nodes were collected, including lymph node involvement, Sum of the largest diameter for multiple lymph nodes (SLD) in each level, extra-nodal neoplastic spread (ENS), and necrosis. Lymph node involvement was determined by multiple criteria [15], including (a) shortest transverse diameter in the largest plane of cervical node > 10 mm and > 5 mm for lateral RPN, and any node seen in the median RPN; (b) central necrosis, extra-nodal neoplastic spread; (c) three or more contiguous and confluent lymph nodes, with shortest transverse diameter > 8 mm. SLD meant sum of the largest diameter in transverse, sagittal, or coronal plane for multiple lymph nodes. And confluent lymph nodes were evaluated as single one.

### Statistical analysis

SPSS 16.0 software package was used for statistical analysis. The distribution of exposure factors between case and control groups was evaluated using *t* test or Wilcoxon rank sum test for quantitative variables, and the chi-square test or Fisher exact test for categorical variables. Receiver operating characteristic (ROC) curve analysis was used to evaluate different cut-off points for SLD to discriminate case or control group. Binary logistic regression was used to estimate the correlation between multiple exposure factors and the PLNM. With a two-sided test, p value of <0.05 was considered statistically significant.

## Results

Median age of the patients in this study was 42 (range, 9–67) years. All patients had WHO type II or III disease. And 41/63 (65.1 %) patients were T3-4 disease. 47/63 (74.6 %) patients came to hospital because of a chief complaint of the mass in neck. Table 1 showed the clinical features of patients. And there were no significant difference in clinical features between the case and control groups.

**Table 1:** Clinical features of patients in case and control groups

Clinical features	Total No. (%)	Cases (19 pts)	Controls (44 pts)^a^	*p* value*
Age				0.840
Median	42.0	46.0	42.0	
Range	9-67	9-66	16-67	
Sex				1.000
Male	49 (77.8)	15	34	
Female	14 (22.2)	4	10	
Histology				0.979
Differentiated	33 (52.4)	10	23	
Undifferentiated	30 (47.6)	9	21	
T stage				0.346
T1-2	22 (34.9)	5	17	
T3-4	41 (65.1)	14	27	
Mass in neck (C.C.)^b^				0.142
Yes	47 (74.6)	17	30	
No	16 (25.4)	2	14	

^a^Control group: N2-3 NPC patients without periparotid lymph node metastasis

^b^Mass in neck (C.C.), a chief complaint of the mass in neck

*p < 0.05

### Parotid lymph nodes metastasis

**Fig. 1:**
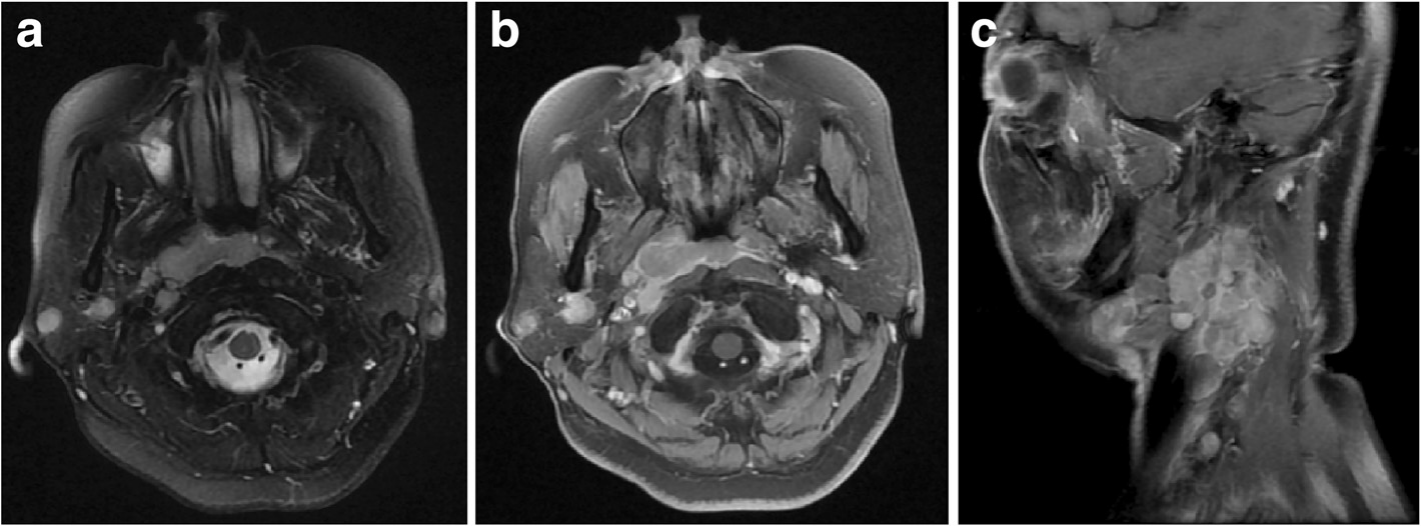
One NPC patient with superficial and deep intraparotid nodes metastasis. **a**. Transverse T2WI; **b**. Transverse T1WI with contrast enhancement; **c**. Sagittal T1WI with contrast enhancement in the ipsilateral neck

Totally, there were 48 positive lymph nodes found in the 22 cases of PLNM. Of the 48 positive lymph nodes, 21 nodes were located in superficial intraparotid, 13 nodes in subparotid, 11 nodes in deep intraparotid, and 3 nodes in pre-auricular area, respectively. The median of largest transverse diameter of nodes in parotid was 1.0 cm (range, 0.4–3.1 cm). And the median of shortest transverse diameter in the largest plane of parotid node was 0.7 cm (range, 0.4–2.0 cm). There were 10 cases of ENS and 8 cases of necrosis in metastatic parotid nodes. Fig. 1 showed one NPC patient with parotid lymph nodes metastasis, and the extensive lymphadenopathy can be seen in ipsilateral neck.

### Regional lymph nodes metastasis in case and control groups (Table 2)

**Table 2:** Ipsilateral neck lymph nodes metastasis in case and control groups

Variables	Total No. (%)	Cases(22 sides)	Controls(44 sides)^a^	*p* value*
RPN^b^				
Involvement	53 (80.3)	20	33	0.229
SLD^c^	1.8 (0-5.5)	2.4(0-5.5)	1.5(0-4.7)	0.106
ENS^d^	43 (65.2)	17	26	0.144
Necrosis	20 (30.3)	6	14	0.705
Level II				
Involvement	66 (100)	22	44	-
SLD^c^	4.5 (1.0-14.7)	6.0(1.5-14.7)	3.6(1.0-9.0)	0.003*
SLD ≥ 5.0 cm	28 (42.4)	16	12	<0.001*
ENS^d^	59 (89.4)	21	38	0.409
Necrosis	44 (66.7)	19	25	0.016*
Level III				
Involvement	45 (68.2)	16	29	0.575
SLD^c^	0.9 (0-7.2)	1.0(0-7.2)	0.8(0-3.8)	0.211
ENS^d^	33 (50.0)	10	23	0.602
Necrosis	14 (21.2)	4	10	0.915
Level IV				
Involvement	21 (31.8)	10	11	0.093
SLD^c^	0.0 (0-6.9)	0.0(0-6.9)	0.0(0-2.0)	0.179
ENS^d^	15 (22.7)	7	8	0.213
Necrosis	4 (6.1)	2	2	0.596
Level Va/b				
Involvement	19 (28.8)	10	9	0.034*
SLD^c^	0.0 (0-9.3)	0.0(0-9.3)	0.0(0-2.2)	0.299
ENS^d^	6 (9.1)	4	2	0.167
Necrosis	4 (6.1)	3	1	0.104
Rare-neck area involvement^e^				
	18 (27.3)	12	6	<0.001*
No. of involved levels^f^				
Median	2 .0(1-5)	3.5(1-5)	2.0(1-5)	0.045*
≥ 4 levels	17 (25.8)	11	6	0.001*

^a^Control group: The ipsilateral neck with primary tumor center or the side with heavier neck disease in the N2-3 NPC patients without PLNM

^b^RPN: Retropharyngeal lymph node

^c^SLD: Sum of the largest diameter for multiple lymph nodes in different neck levels. Median and range were listed in table

^d^ENS: Extra-nodal neoplastic spread

^e^Rare neck areas including level Ia/b, level Vc, and level VI in this study

^f^No. of involved levels, when RPN was excluded and rare-neck areas were counted as one level

*p < 0.05

Involvement of RPN was found in 53/66 (80.3 %) sides. ENS and necrosis in RPN were found in 43/66 (65.2 %) and 20/66 (30.3 %) sides, respectively. RPN involvement, ENS, necrosis, and SLD showed no significant difference in case and control group.

Level II contained IIa and IIb subareas. All patients in this study were involved with level II. ENS and necrosis in level II were found in 59/66 (89.4 %) and 44/66 (66.7 %) sides. And level II necrosis in case group was significantly higher than that in control group (86.4 % *vs*. 56.8 %, *p* = 0.016). The median of SLD in level II was 4.5 cm (range, 1.0–14.7 cm) in the sets. And the SLD of level II in case group was larger than that in control group (median, 6.0 *vs*. 3.6 cm, *p* = 0.003).

Level III involvement, ENS, and necrosis was found in 45/66 (68.2 %), 33/66 (50.0 %), and 14/66 (21.2 %) sides in this study, respectively. The median of SLD in level III was 0.9 cm (range, 0–7.2 cm). And no significant difference in level III involvement, ENS, necrosis, and SLD was found in case and control groups.

Level IV involvement, ENS, and necrosis were found in 21/66 (31.8 %), 15/66 (22.7 %), 4/66 (6.1 %) sides in this study, respectively. And no significant difference in level IV involvement, ENS, necrosis, and SLD was found in case and control groups.

Level Va/b involvement was found in 19/66 (28.8 %) sides in this study. And the rate of level Va/b involvement was higher in case group than that in control group (45.5 % *vs*. 20.5 %, *p* = 0.034). There was no significant difference in ENS, necrosis, and SLD in level Va/b between the two groups.

Rare-neck areas in this study contained level Ia/b, Vc and VIa/b. And level Ia/b, Vc and VIa/b involvement were found in 6, 8, and 2 sides in case group, respectively. The rate of rare-neck areas involvement was higher in case group than that in control group (54.5 % *vs*. 13.6 %, *p* < 0.001).

### Multivariate analysis

ROC curve was used to determine the most suitable cut-off SLD in level II (AUC =0.73, *p* = 0.003). 5.0 cm (≥5.0 *vs*. <5.0) was selected as the cut-off point with sensitivity of 72.7 % and specificity of 72.7 %. Binary logistic regression model was used to analyze the high-risk factors of PLNM in multivariate analysis. When SLD ≥5.0 cm in level II, necrosis in level II, involvement in level Va/b, and involvement in rare-neck areas were included into analysis, only SLD ≥5.0 cm in level II (OR = 4.11, 95 % CI 1.15-15.73, *p* = 0.030) and rare-neck areas involvement (OR = 3.95, 95 % CI 1.03-15.09, *p* = 0.045) were associated with PLNM in NPC patients (Table 3).

**Table 3:** Multivariate analysis of PLNM

Variables	β value	OR	95 % CI		*p* value*
			Lower	Upper	
Level II SLD^a^ ≥ 5.0 cm	1.41	4.11	1.15	14.73	0.030*
Level II necrosis	0.85	2.34	0.51	10.87	0.277
Level Va/b	0.45	1.56	0.25	9.59	0.631
Rare neck areas^b^	1.35	3.95	1.03	15.09	0.045*

^a^Level II SLD, sum of the longest diameter for multiple lymph nodes in level II

^b^Rare neck areas including level Ia/b, level Vc, and level VI in this study

*p < 0.05

## Discussion

PLNM was thought to be an uncommon event in NPC patients. It has been reported that the incidence of PLNM was 0.6 % ~ 3.0 % in whole NPC patients [3–7, 16]. And similarly, the incidence of PLNM was 1.8 % in whole patients and was 3.3 % in N2-3 NPC patients in this study. According to the size of metastatic nodes in parotid, it should be highly suspected that any single lymph node of parotid with largest transverse diameter ≥ 0.5 cm or multiple nodes with borderline size in NPC patients who were simultaneously suffered from heavy lymph node invasion in ipsilateral neck. And the nodes in parotid should be confirmed by ultrasonographically guided FNA or CT-guided biopsy. In this study, 48 lymph nodes in 22 parotids were found to be positive, and superficial intraparotid was the most frequently involved subarea (43.8 %). 10 (45.5 %) parotids showed multiple subareas invasion. Therefore, if there were PLNMs, the whole parotid may be supposed to be in target volume.

Retropharyngeal nodes were the first echelon of nodal metastases in NPC and the incidence of RPN involvement was 75.1–94.0 % in node-positive NPC [3–7, 16]. However, RPN involvement, ENS, necrosis, and SLD showed no significant difference between the two groups. Our findings seemed not to support the theory that tumor can reach the parotid gland directly via the RPNs [17]. In fact, except for parapharyngeal space involvement, there were no correlations between RPN metastasis and surrounding invasions [18]. Direct lymphatic spread from RPN to parotid tissue was questioned.

Like RPNs, level II was also proved to be the first-echelon nodal metastasis for NPC. And the previously reported incidence of level II involvement was 75.1–95.5 % in node-positive NPC patients [3–6]. Our finding showed 100 % involvement in level II in both groups. Level II involvement alone was not enough to predict the PLNM. The parotid gland contains an extensive lymphatic capillary plexus. However, patterns of metastasis to the parotid nodes were hardly distinguished from a recognized drainage area and from widespread metastatic disease in the neck and involvement by retrograde extension [19]. Anatomically, lymphatics of parotid drained along the retromandibular vein to superficial nodes along the outer surface of the sternocleidomastoid muscle, and then partially drained into upper nodes of the deep cervical chain [20]. In 2009, Pan et al. [21] showed drawings of the lymphatic pathways from nasopharynx. Lymphatic vessels from nasopharynx drained in two general directions, lateral pharyngeal and retropharyngeal directions. The former one descended along the pharyngeal wall in the parapharyngeal fat tissue to reach its first tier lymph node, level II node, which was situated on the lateral side of the external carotid artery [21]. No direct lymphatic drainage was found from nasopharynx to parotid area in Pan’s study. However, we suppose that level II nodes that connected superficial and deep cervical nodes may play crucial role in PLNM from retrograde extension. Our findings showed clinical evidences that PLNM was closely correlated with SLD and necrosis of level II. NPC patients with heavy disease in level II may cause the blockage of the normal routes of lymphatic drainage and induce retrograde tumor spread to parotid lymph nodes. SLD ≥5.0 cm and necrosis may be indicators of heavy disease of level II and be potential high-risk factors for PLNM.

Level III, IV, and Va/b represented second-echelon of nodal metastases in NPC with the incidence of involvement of 44.9 %, 11.2 %, and 26.7 %, respectively [1]. Lymph node metastases progressed in an orderly way and skip metastases were rarely found [3, 6]. Our findings showed that the incidence of level III, IV, and Va/b involvement in case group were 72.7 %, 45.5 %, and 45.5 %, respectively. Caudal (level III/IV) and posterior (level V) lymphatic spreads were higher involved in our study than that in previous results [3–6]. Although level III involvement showed no significant difference in case and control groups, Level IV and Va/b involvement appeared to be marginal and significant difference, respectively. Previous studies postulated that widespread metastatic disease in the neck may count for PLNM, but no evidence listed [6, 7]. Our results showed that the number of involved levels was correlated with PLNM and that multiple levels (≥4 levels) involvement may be a potential high-risk factor for PLNM (Table 2).

Rare-neck areas in this study were defined as level Ia/b, Vc, and VIa/b, which all belonged to third-echelon of nodal metastases in NPC [1]. The incidence of rare-neck areas involvement was higher in case group than that in control group (54.5 % *vs*. 13.6 %, *p* < 0.001). 3.1–4.3 % cases with level Ib involvement has been reported in previous study and all patients were simultaneously accompanied with level II lymphadenopathy [3, 16]. Wang reported 1.8 % level Vc involvement in N-positive NPC patients. And level Vc metastasis was associated with multiple levels involvement and the total number of positive nodes was always more than seven. To a large extent, 3th-echelon lymph nodes metastasis in NPC was associated with extensively adjacent neck disease. Parotid lymph node was also regarded as the third-echelon of draining nodes in NPC [1]. Probably because it was a surrogate marker of extensive ipsilateral nodal disease, the rare-neck areas involvement was closely correlated with PLNM in this study.

Multivariate analysis showed that SLD ≥5.0 cm in level II (OR = 4.11, *p* = 0.030) and rare-neck areas involvement (OR = 3.95, *p* = 0.045) were independent high-risk factors of PLNM. Any patients with extensive tumor invasion and suspected blockage of lymphatic drainage should be emphasized to conduct parotid detection. In 2008, Cannon reported two cases of periparotid recurrence in NPC patients after definitive IMRT [22]. Both patients had small subclinical periparotid nodules and multilevel nodal disease in ipsilateral neck in pretreatment imaging. The two relapsed nodes were both found in superficial intraparotid that was situated outside of the field and received doses less than 20Gy. In 2003, Chao et al. reported 1 % marginal failure in the region adjacent to the spared parotid gland following IMRT in head and neck cancer [23], but no recommendation has been proposed pertaining to prevention of periparotid failure. Our findings in this study suggested that prophylactic irradiation to the whole or part of the ipsilateral parotid may be necessary for NPC patients with high-risk factors of PLNM.

High-risk factors of PLNM have been discussed for the first time in this study. However, the results of this study should be interpreted with caution, as this was a case-control study of a small sample size. Although periparotid failure was inferred to be related to parotid-sparing IMRT, failure analysis was hard to carry out. Further investigations of large sample from multiple centers may be warranted.

## Conclusion

To summarize, PLNM was an uncommon event and the incidence was 1.8 % in NPC patients. Any enlarged single lymph node of parotid with largest transverse diameter ≥ 0.5 cm or multiple nodes with borderline size in NPC patients who were simultaneously companied with extensive ipsilateral nodal disease should be further detected. SLD ≥5.0 cm in level II and involvement in rare-neck areas may be potentially high-risk factors for PLNM in NPC patients. Sparing parotid in IMRT was not recommended for NPC patients with high risks of PLNM.

## Abbreviations

AJCC: American Joint Committee on Cancer
AUC: Area under curve
ENS: Extra-nodal neoplastic spread
FNA: Fine needle aspiration
NPC: Nasopharyngeal carcinoma
PLNM: Parotid lymph node metastasis
ROC: Receiver operating characteristic curve
RPN: Retropharyngeal lymph node
SLD: Sum of the longest diameter for multiple lymph nodes
